# Keratinocyte-derived I**κ**B**ζ** drives psoriasis and associated systemic inflammation

**DOI:** 10.1172/jci.insight.130835

**Published:** 2019-11-14

**Authors:** Sebastian Lorscheid, Anne Müller, Jessica Löffler, Claudia Resch, Philip Bucher, Florian C. Kurschus, Ari Waisman, Knut Schäkel, Stephan Hailfinger, Klaus Schulze-Osthoff, Daniela Kramer

**Affiliations:** 1 Interfaculty Institute for Biochemistry, University of Tübingen, Tübingen, Germany.; 2 Department of Dermatology, Heidelberg University Hospital, Heidelberg, Germany.; 3 Institute for Molecular Medicine, University Medical Center of the Johannes Gutenberg-University Mainz, Mainz, Germany.; 4 Cluster of Excellence iFIT (EXC 2180) “Image-Guided and Functionally Instructed Tumor Therapies,” University of Tübingen, Tübingen, Germany.; 5 German Cancer Consortium and German Cancer Research Center, Heidelberg, Germany.

**Keywords:** Autoimmunity, Dermatology, Autoimmune diseases, Innate immunity, Skin

## Abstract

The transcriptional activator IκBζ is a key regulator of psoriasis, but which cells mediate its pathogenic effect remains unknown. Here we found that IκBζ expression in keratinocytes triggers not only skin lesions but also systemic inflammation in mouse psoriasis models. Specific depletion of IκBζ in keratinocytes was sufficient to suppress the induction of imiquimod- or IL-36–mediated psoriasis. Moreover, IκBζ ablation in keratinocytes prevented the onset of psoriatic lesions and systemic inflammation in keratinocyte-specific IL-17A–transgenic mice. Mechanistically, this psoriasis protection was mediated by IκBζ deficiency in keratinocytes abrogating the induction of specific proinflammatory target genes, including *Cxcl5*, *Cxcl2*, *Csf2*, and *Csf3*, in response to IL-17A or IL-36. These IκBζ-dependent genes trigger the generation and recruitment of neutrophils and monocytes that are needed for skin inflammation. Consequently, our data uncover a surprisingly pivotal role of keratinocytes and keratinocyte-derived IκBζ as key mediators of psoriasis and psoriasis-related systemic inflammation.

## Abstract 2

Deletion of IκBζ in keratinocytes is sufficient to abrogate psoriasis induction in mouse models due to changes in transcription of keratinocyte-derived chemo- and cytokines.

## Graphical abstract

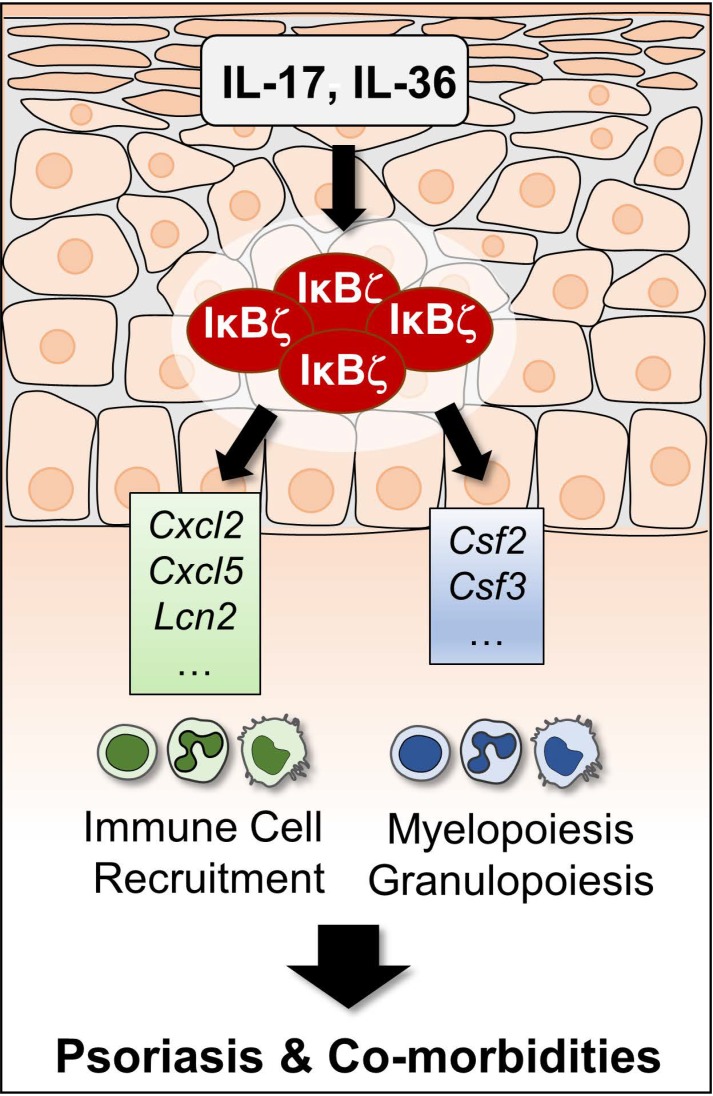

## Introduction

Psoriasis constitutes a common autoinflammatory disease of the skin, which is characterized by keratinocyte hyperproliferation as well as massive infiltration of neutrophils, macrophages, and IL-17A–expressing T cells (1). Moreover, psoriasis is often associated with systemic inflammation and comorbidities, such as arthritis, cardiovascular disease, and liver inflammation because of the generation, mobilization, and tissue infiltration of proinflammatory neutrophils and monocytes (1–3). On the molecular level, chronic IL-17A/IL-23 signaling was found to drive the development of the most common subtype of psoriasis, psoriasis vulgaris (4). Another severe subtype, called generalized pustular psoriasis, was found to result from an overactive IL-36 pathway (5). In agreement, neutralizing antibodies against IL-17A and IL-23 represent effective state-of-the-art therapies for the treatment of patients with psoriasis, whereas antagonizing IL-36 receptor antibodies are currently being investigated as a new therapy approach for psoriasis (6).

Interestingly, it has recently been revealed that expression of IκBζ, encoded by *NFKBIZ*, is upregulated in psoriatic lesions, while global IκBζ deficiency in mice completely protects against imiquimod-mediated (IMQ-mediated) or IL-36α–mediated psoriasis (7, 8). IκBζ belongs to the family of atypical IκBs and constitutes a cofactor of the transcription factor NF-κB that is inducibly expressed in the nucleus, leading to the activation or repression of a selective subset of NF-κB target genes (9–12). Although the downstream mechanism of IκBζ function remains unclear, it is suggested that IκBζ recruits epigenetic factors to the promoter region of its target genes, thereby modulating gene expression (13, 14). Induction of IκBζ and subsequent target gene expression has been investigated in different cell types, such as keratinocytes, macrophages, dendritic cells, and T cells (15). Stimulation of keratinocytes with IL-17A or IL-36 induces IκBζ expression, leading to the induction of several psoriasis-associated target genes, such as *Cxcl8*, *Cxcl2*, *Defb4*, or *S100a9* (7, 8). In T_H_17 cells, IκBζ was found to cooperate with RORγt in regulating the expression of *Il17a*, *Il22*, and *Tnfa* (16). IκBζ can thus regulate inflammatory responses in several cell types. Although IκBζ has emerged as a novel regulator for the pathogenesis of psoriasis, it remains unclear whether IκBζ expression in dendritic cells, macrophages, neutrophils, T cells, or keratinocytes is relevant for its pathogenic effects. Furthermore, whether IκBζ plays a role in psoriasis-related systemic inflammation and the development of comorbidities is unknown.

To address these questions, we generated keratinocyte-specific IκBζ-deficient mice (K14-Cre *Nfkbiz* KO) and investigated IMQ-, IL-36–, and IL-17A–mediated psoriasis induction in these mice. Surprisingly, we found that keratinocyte-specific depletion of IκBζ was sufficient to protect against experimental psoriasis in different mouse models. Targeted gene disruption in keratinocytes prevented the induction of IκBζ-dependent target genes, such as *Cxcl2*, *Cxcl5*, and *Csf3*, which did not impair T cell infiltration but strongly suppressed the tissue recruitment of neutrophils and monocytes. Furthermore, keratinocyte-specific ablation of IκBζ was sufficient to block systemic inflammation and the onset of psoriatic lesions in keratinocyte-specific IL-17A–overexpressing mice (17, 18) by regulating a small but critical subset of IL-17A–responsive genes. Hence, our data identify keratinocyte-derived IκBζ as an essential mediator of IL-17A– and IL-36–dependent gene expression and a main driver of psoriasis pathogenesis. These data reveal a surprisingly pivotal role of proinflammatory gene expression of keratinocytes, which drives not only the development of skin lesions but also psoriasis-related systemic inflammation.

## Results

### Keratinocyte-specific depletion of IκBζ protects against IMQ-induced psoriasis.

Previously, it was shown that global IκBζ-deficient mice are protected against IMQ-induced psoriasis (7), which represents the standard mouse model of psoriasis, reflecting many of the key characteristics of human psoriasis (4). Although IκBζ constitutes a key transcriptional regulator driving the onset of psoriasis, the cell type(s) in which IκBζ is induced to exert its psoriasis-promoting function remains unclear. Because of its function in mediating IL-17A expression in T_H_17 cells as well as its role as a downstream mediator of IL-17A and IL-36 signaling in keratinocytes (7, 8, 16, 19), it is speculated that induction of IκBζ in one of these cell types is responsible for the development of psoriasis. To answer this question, we generated keratinocyte-specific *Nfkbiz*-deficient mice and analyzed IMQ-induced psoriasis. To assess the effects of a keratinocyte-specific depletion of IκBζ, global IκBζ-deficient mice (8) were analyzed in parallel. Global deletion of IκBζ was studied in inducible-KO mice that received tamoxifen (TAM) for 4 consecutive days to induce Cre recombinase activation. Subsequently, IMQ-containing Aldara cream was administered daily on the ears of the mice for 7 days (Supplemental Figure 1A; supplemental material available online with this article; https://doi.org/10.1172/jci.insight.130835DS1).

**Figure 1:**
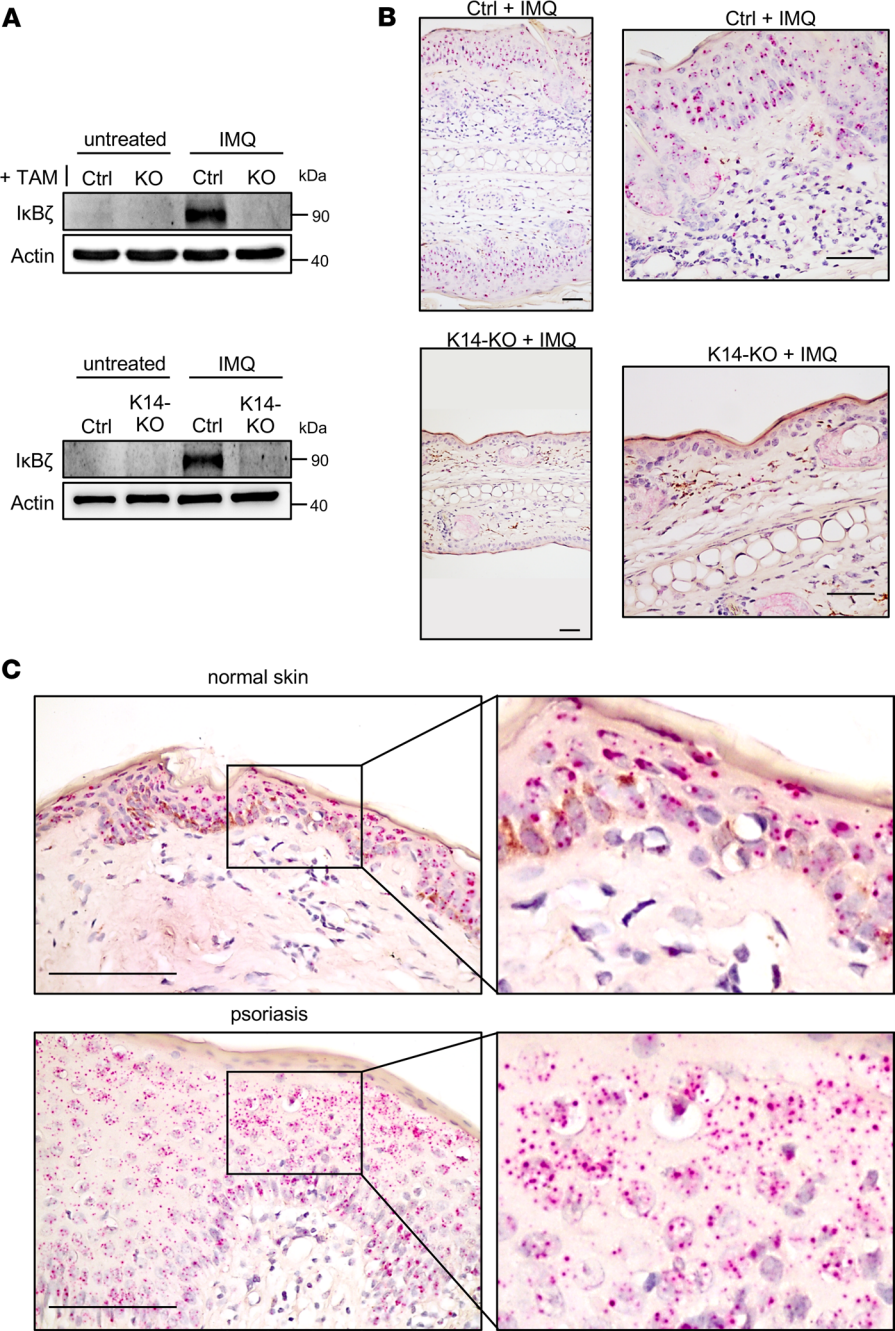
*NFKBIZ* expression in mouse and human skin. (**A**) Induction of IκBζ in whole-skin lysates from untreated and IMQ-treated, TAM-induced global (KO, upper) or keratinocyte-specific (K14-KO, lower) IκBζ-deficient mice at day 7. Actin served as a loading control. (**B**) Predominant localization of *Nfkbiz* in the epidermis of IMQ-treated control mice, which is absent in IMQ-treated K14-KO mice. Scale bars: 40 μm. (**C**) Keratinocyte-specific *NFKBIZ* expression was also detected in normal human skin (upper). As shown by the increased number of red dots, *NFKBIZ* expression was elevated in human psoriatic skin lesions (lower). Following deparaffinization tissue sections were hybridized with mouse or human *NFKBIZ*-specific RNAScope target probe sets consisting of multiple tandem short oligonucleotides. *NFKBIZ* mRNAs were visualized as dots, with each dot representing a single RNA transcript. Right images show sections of the pictures on the left at a higher magnification. Scale bars: 100 μm.

In agreement with previous analyses (7), IκBζ was effectively induced in the skin of IMQ-treated control mice, whereas no expression could be detected in IMQ-treated ears of TAM-treated global KO mice (Figure 1A). Surprisingly, IκBζ expression was also completely absent in skin tissue of IMQ-treated mice lacking IκBζ specifically in keratinocytes (K14-KO). Accordingly, *Nfkbiz* mRNA was expressed mainly in the epidermis but only rarely in the infiltrating immune cells of the dermis, as detected by RNAScope in situ hybridization using IMQ-treated ears (Figure 1B). Furthermore, we detected an epidermis-restricted expression pattern of *NFKBIZ* mRNA in human skin biopsies, which was increased in psoriatic lesions compared with normal skin (Figure 1C). Thus, *Nfkbiz* mRNA levels seem to be expressed predominantly in the keratinocyte compartment during psoriasis.

**Figure 2:**
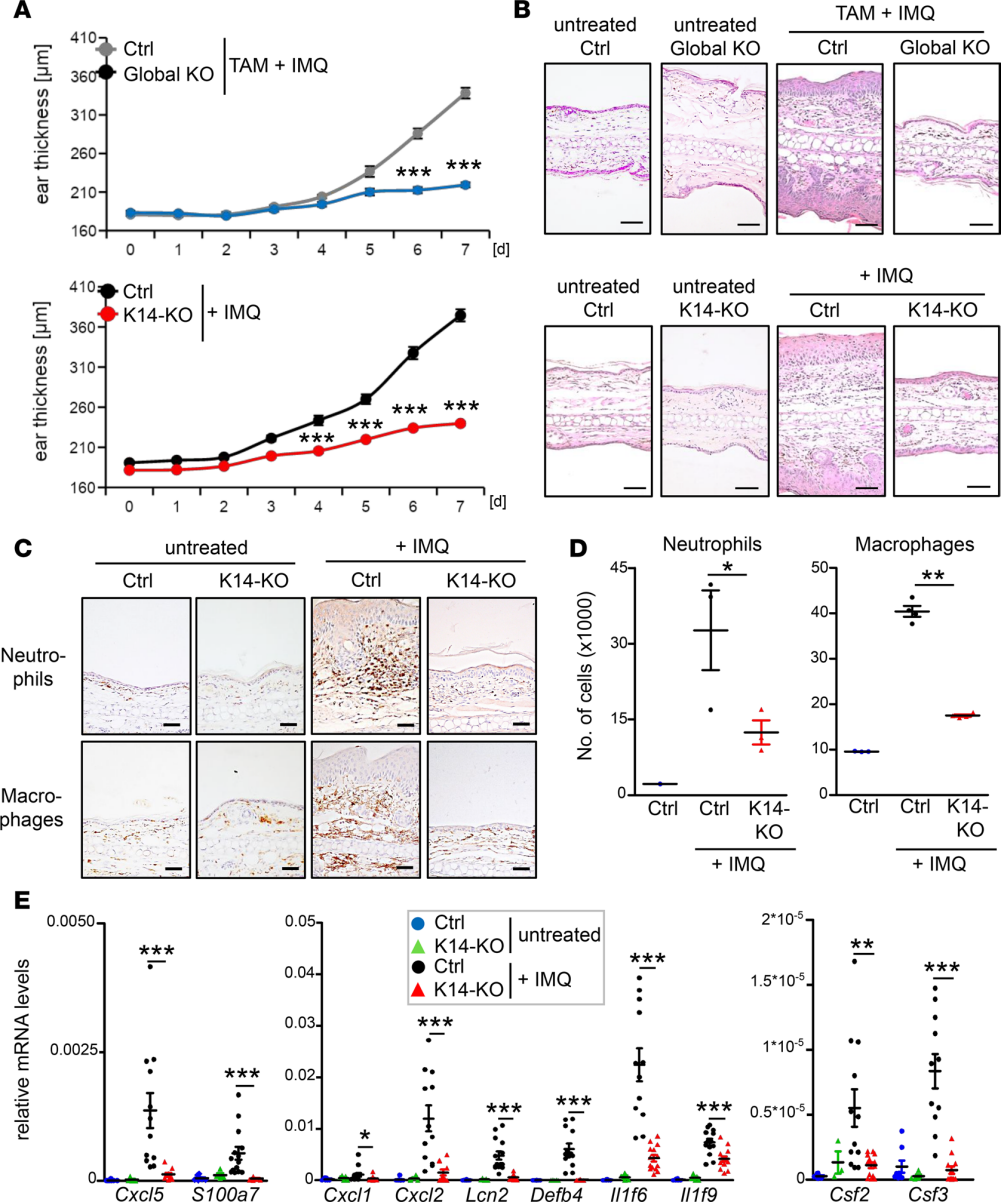
Keratinocyte-specific deletion of IκBζ protects against IMQ-induced psoriasis. All analyses were performed after 7 days of IMQ treatment. (**A**) Measurement of the ear thickness from global and K14-KO mice during 7 days of daily IMQ treatment. *Top:* TAM-treated control (Ctrl) and global *Nfkbiz*-KO mice. *n* = 6. *Bottom:* Control and K14-*Nfkbiz*–KO mice. *n* = 20. (**B**) H&E staining from ears of untreated and IMQ-treated mice. Scale bars: 100 μm. (**C**) IHC detection of infiltrating neutrophils (marker myeloperoxidase [MPO]) and macrophages (marker F4/80) in untreated and IMQ-treated K14-KO mice. Scale bars: 50 μm. (**D**) Quantification of infiltrating neutrophils (Ly6G^+^) and macrophages (F4/80^+^) by flow cytometry analysis. Depicted is the relative number of infiltrating immune cells from whole ears of untreated and IMQ-treated mice. *n* = 3–4 ± SEM. (**E**) Gene expression analysis of untreated and IMQ-treated control and K14-KO mice. Relative mRNA expression of psoriasis-related genes was analyzed from 4–14 ear skin samples per group ± SEM and normalized to the reference gene *Actin*. *P* values were calculated using 2-tailed Student’s *t* test (*P < 0.05, **P < 0.01, and ***P < 0.001).

Importantly, whereas IMQ treatment of control mice led to the typical alterations of psoriasis, K14-KO mice were completely protected against ear swelling, keratinocyte hyperproliferation, and immune cell infiltration, which was also entirely absent in global KO mice (Figure 2, A and B). Detailed analysis of the immune cell infiltrates revealed a strong reduction in neutrophil and macrophage recruitment in K14-IκBζ–deficient mice (Figure 2, C and D, Supplemental Figure 1B), which was reduced to a similar extent as in IMQ-treated global IκBζ-deficient mice (Supplemental Figure 1C). Accordingly, expression of several genes encoding chemokines involved in neutrophil and macrophage recruitment, such as *Cxcl5*, *Cxcl1*, and *Cxcl2*, and antimicrobial proteins, such as *Lcn2* or *Defb4*, was strongly downregulated in the skin of keratinocyte-specific and global KO mice (Figure 2E and Supplemental Figure 1D). Of note, a similarly strong reduction in ear swelling and expression of psoriasis-relevant genes was detectable when IκBζ was depleted after establishment of IMQ-induced skin inflammation (Supplemental Figure 1, E–H). Thus, IκBζ represents a key factor in the skin that is needed not only for the induction but also for the progression of IMQ-induced psoriasis-like skin inflammation.

### Infiltration of IL-17A–producing γδ T cells is not impaired in keratinocyte-specific IκBζ-KO mice after IMQ treatment.

**Figure 3:**
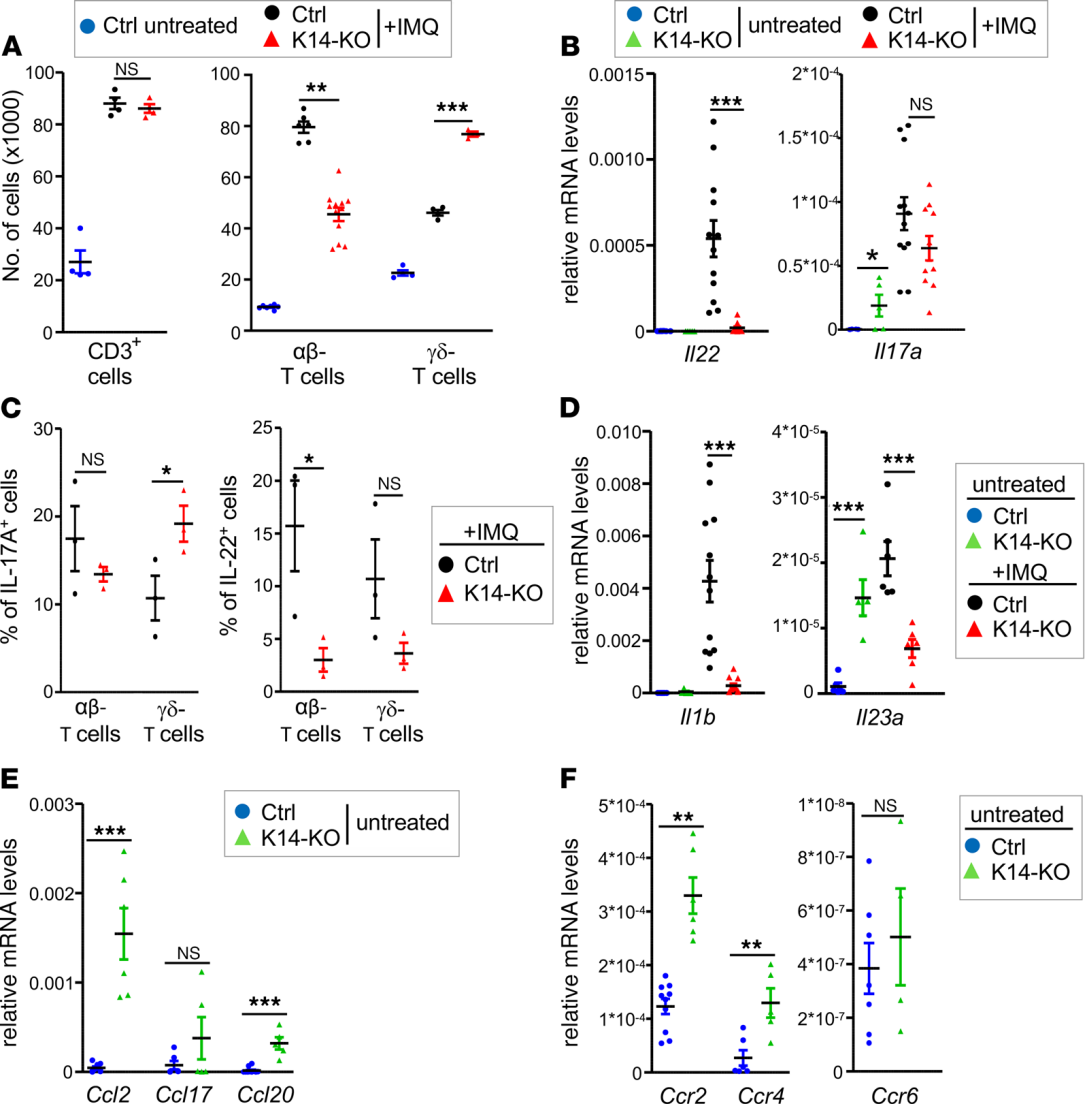
Analysis of skin-infiltrating T cells in IMQ-treated K14-IκBζ–KO mice. All analyses were performed after 7 days of IMQ treatment. (**A**) Flow cytometry analysis of T cell subsets in the ears of IMQ-treated Ctrl and K14-KO mice. T cell subsets were detected as CD45^+^ and CD3^+^, αβTCR^+^, or γδTCR^+^ cells. Single data points derive from 2 ears. Shown is the mean of 4–12 mice per group ± SEM. (**B**) Gene expression analysis of *Il22* and *Il17a* in skin tissue of untreated and IMQ-treated control and K14-KO mice, normalized to the reference gene *Actin*. *n* = 4–14 ± SEM. (**C**) Determination of the percentage of IL-17A– and IL-22–producing αβ and γδ T cells in IMQ-treated control and K14-KO mice. After fixation and permeabilization, cells were gated as in **A**, except for an additional gating on either IL-17A^+^ or IL-22^+^ cells. *n* = 3 ± SEM. (**D**) Gene expression analysis of *Il1b* and *Il23a* in untreated and IMQ-treated mice, similar as in **B**. (**E**) mRNA levels of *Ccl2*, *Ccl17*, and *Ccl20* in untreated mice, similar as in **B**. (**F**) mRNA levels of *Ccr2*, *Ccr4*, and *Ccr6* in the skin of untreated control and K14-KO mice, similar as in **B**. *n* = 5–14 ± SEM. *P* values were calculated using 2-tailed Student’s *t* test (*P < 0.05, **P < 0.01, and ***P < 0.001).

Whereas IMQ-induced neutrophil and macrophage infiltration was strongly reduced in IMQ-treated K14-KO mice, infiltration of CD3^+^ and especially γδ T cells was surprisingly not impaired in the KO mice compared to control mice (Figure 3A and Supplemental Figure 2A). Moreover, whereas the T cell–associated cytokine *Il22* was significantly downregulated by keratinocyte-restricted IκBζ deficiency, *Il17a* expression remained elevated in the skin of IMQ-treated K14-KO mice (Figure 3B). Further analysis revealed that IL-17A and IL-22 expression derived from both infiltrating αβ and γδ T cells in control and K14-IκBζ–KO mice, while the frequency of IL-17A–expressing γδ T cells especially was increased in IMQ-treated K14-IκBζ–KO mice (Figure 3C and Supplemental Figure 2B).

IL-1β and IL-23 are induced during IMQ-induced psoriasis and trigger the expression of *Il17a* from γδ T cells (20). Surprisingly, however, *Il1b* and *Il23a* expression were significantly downregulated in IMQ-treated K14-KO mice and therefore could not account for the elevated numbers of IL-17A–expressing γδ T cells in these mice (Figure 3D). Instead, we already detected an elevated expression of *Il17a* and *Il23a* along with an expansion of γδ T cells in the skin of untreated K14-KO mice (Figure 3B and Supplemental Figure 2C). This was possibly due to an increased expression of *Il7* and *Il15* in the skin of untreated K14-KO mice (Supplemental Figure 2D), which are both cytokines known to regulate tissue homeostasis of γδ T cells (21, 22). These findings agree with a previous report detecting an expansion of T cells in the skin of global *Nfkbiz*-deficient mice because of a changed microbiome (23). Thus, we suggest that the elevated numbers of IL-17A–expressing γδ T cells in keratinocyte-specific IκBζ KO mice are independent of IMQ treatment and possibly due to a changed microbiome.

To further explore the mechanism for increased γδ T cell numbers in the skin of untreated K14-KO mice, we analyzed the expression of T cell–associated chemokines and their receptors. Skin-infiltrating T cells can express Ccr2, Ccr4, or Ccr6, whereas their corresponding ligands, Ccl2, Ccl20, and Ccl17, are secreted by keratinocytes or endothelial cells (24), thus triggering the recruitment of different lymphocyte subsets into the inflamed skin. Our analyses revealed that in particular *Ccl2* and, to a minor extent, *Ccl17* and *Ccl20* were overexpressed in the skin of untreated IκBζ-deficient K14-KO mice compared with control mice (Figure 3E). Moreover, the expression levels of *Ccr2*, *Ccr4*, and *Ccr6* were also upregulated in the skin of K14-KO mice (Figure 3F). In agreement with previous reports (25, 26), these results indicate that increased signaling via the Ccl2/Ccr2 axis, and eventually via Ccl17/Ccr4 and Ccl20/Ccr6, could contribute to the increased presence of γδ T cells in the skin of K14-KO mice. Importantly, however, despite the increased numbers of IL-17A–expressing γδ T cells in the skin, K14-KO mice were strongly protected against IMQ-induced keratinocyte hyperproliferation, macrophage and neutrophil recruitment, as well as ear swelling.

### Lack of IκBζ expression in keratinocytes protects against IL-36–induced dermatitis.

**Figure 4:**
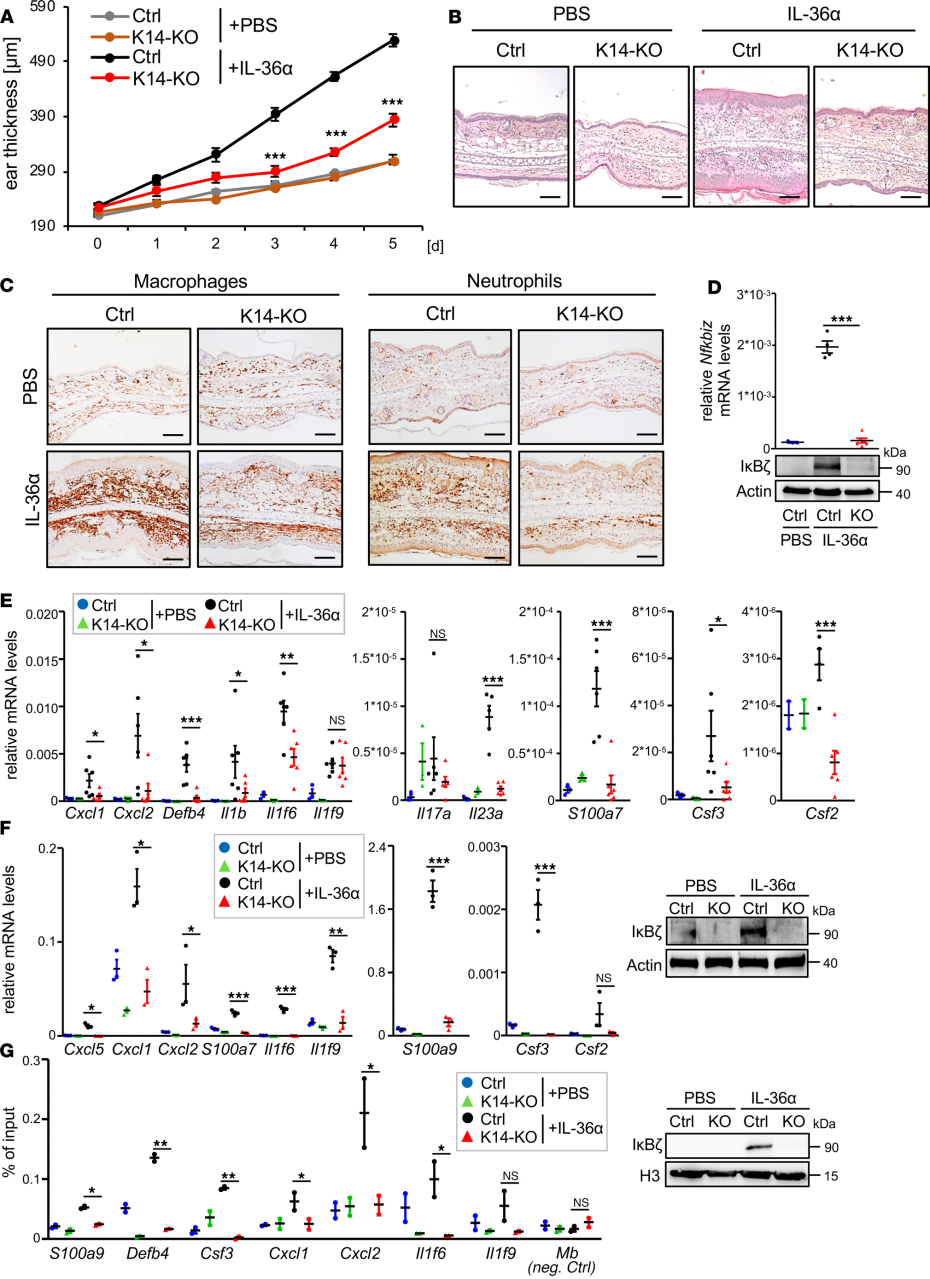
Deletion of IκBζ in keratinocytes protects against IL-36–induced dermatitis. (**A**) Ear thickness of control Ctrl and K14-Cre *Nfkbiz*-KO mice that were treated with intradermal injections of PBS as control or 1 μg recombinant murine IL-36α for 5 consecutive days. *n* = 6, ± SEM. (**B**) H&E staining of ears from control and K14-KO mice at day 6. Scale bars: 100 μm. (**C**) IHC staining of macrophages (F4/80 staining) and neutrophils (MPO staining) in control and IL-36α–treated mice at day 6. Scale bars: 100 μm. (**D**) IκBζ mRNA and protein levels in IL-36α–treated ear skin samples. (**E**) Psoriasis-related gene expression in the ears of IL-36α–treated control and K14-KO mice with mean ± SEM from 2 to 3 PBS-treated and 6 IL-36α–treated animals per group. (**F**) *Left:* Gene expression in IL-36α–treated murine keratinocytes (mKC). Keratinocytes from 3 Ctrl and 3 K14-KO mice were isolated from the tails and grown to confluence. Upon treatment with 100 ng/mL IL-36α for 1.5 hours, cells were harvested and analyzed for gene expression. Relative mRNA levels were normalized to *Actin*. *Right:* Immunostaining of IκBζ in IL-36α–treated mKC. Control and KO cells were treated for 24 hours with 100 ng/mL IL-36α. (**G**) Detection of IκBζ binding to the promoters of psoriasis-related genes in keratinocytes. *Left:* ChIP of IκBζ or IgG as control was performed in untreated and IL-36α–treated mKC (treatment: 1.5 hours 100 ng/mL IL-36α). Shown is the fold enrichment of anti-IκBζ binding over IgG. The promoter region of myoglobulin (*Mb*) served as an internal negative control. *n* = 2 per group. *Right:* Detection of IκBζ levels in the chromatin fraction as input control. *P* values were calculated using 2-tailed Student’s *t* test (*P < 0.05, **P < 0.01, ***P < 0.001).

IMQ is a TLR7/8 agonist, initially triggering the activation of dendritic cells, macrophages, and neutrophils in the skin. Because human psoriasis is believed to develop from aberrant, chronic signaling in keratinocytes, IMQ-induced psoriasis might not properly reflect an upstream function of IκBζ in keratinocytes. For this reason, we investigated IL-36–mediated psoriasis in mice lacking IκBζ in keratinocytes because IL-36 is believed to trigger psoriasis through chronic activation of keratinocytes. As shown before (8), intradermal injections of biologically active IL-36α for 5 consecutive days induced psoriasis-like dermatitis in control mice, including keratinocyte hyperproliferation as well as massive infiltration of neutrophils, macrophages, and T cells (Figure 4, A–C). In line with the IMQ model, keratinocyte-specific *Nfkbiz*-KO mice were completely protected against IL-36–induced ear swelling, hyperkeratosis (Figure 4, A and B), as well as infiltration of neutrophils and macrophages (Figure 4C).

In line with the observations upon IMQ treatment, intradermal administration of IL-36α led to the induction of IκBζ mRNA and protein expression in the skin of control mice, whereas IκBζ could not be detected in IL-36α–treated K14-KO mice (Figure 4D). Similar to the IMQ model, skin samples of IL-36α–treated, keratinocyte-specific IκBζ-deficient mice did not show induction of genes involved in the recruitment and activation of neutrophils and macrophages, such as *Cxcl5*, *Cxcl2*, or *Csf3* (Figure 4E). Moreover, the same set of target genes could not be induced by IL-36α treatment of keratinocyte cultures isolated from IκBζ-KO mice (Figure 4F). To show that IκBζ directly regulates the induction of psoriasis-associated genes, we performed ChIP analysis of IκBζ in IL-36–stimulated control or IκBζ-KO keratinocytes. We found that, upon IL-36 stimulation, IκBζ was actively recruited to the promoter regions of its target genes, including genes encoding for the chemokines *Csf3*, *Cxcl1*, and *Cxcl2* and the psoriasis-associated antimicrobial proteins *Defb4* and *S100a9* (Figure 4G). Thus, keratinocyte-derived IκBζ mediates IMQ- and IL-36–induced psoriasis through the direct transcriptional activation of several psoriasis-associated, proinflammatory genes in keratinocytes.

### Keratinocyte-specific depletion of IκBζ prevents IL-17A–dependent psoriasis.

**Figure 5:**
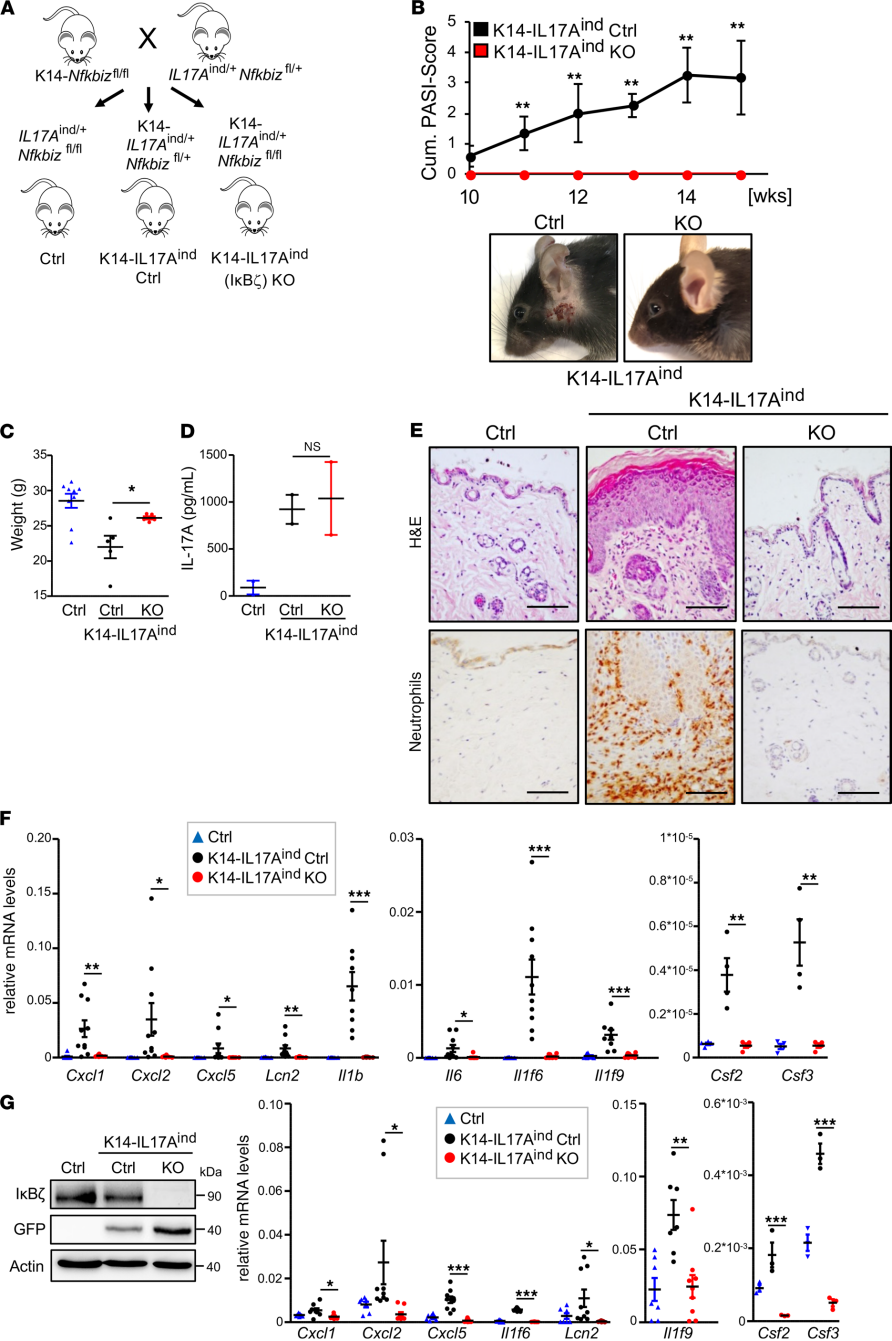
KO of IκBζ in keratinocytes protects against IL-17A–mediated psoriasis. All analyses (panels **C**–**G**) were performed with 15-week-old male mice. (**A**) Breeding scheme for the generation of keratinocyte-specific *Il17a*-overexpressing mice with either heterozygous (K14-IL17A^ind^ Ctrl) or homozygous (K14-IL17A^ind^–KO) deletion of *Nfkbiz*. (**B**) Disease progression in K14-IL17A^ind^ mice in the presence or absence of IκBζ. Shown is the cumulative Psoriasis Area and Severity Index (PASI) score accounting for skin lesions, erythema, scaling, and the percentage of the affected skin area. 0 = no phenotype, healthy. 5 = severely affected. *n* = 6. *Bottom:* pictures of mice. (**C**) Body weight of control and IL17A^ind^ mice in the presence and absence of IκBζ. *n* = 5–9 mice per group ± SEM. (**D**) Serum levels of IL-17A. *n* = 2. (**E**) H&E staining of the back skin and IHC detection of MPO as a marker for neutrophil infiltration. Scale bar: 100 μm. (**F**) Gene expression of psoriasis-related genes in the skin from 4 to 6 animals per group (mean ± SEM). Depicted are relative mRNA levels normalized to *Actin*. (**G**) *Left:* Immunoblot control of *Il17a* overexpression and IκBζ deletion in lysates from isolated mKCs. Because the *Il17a* expression cassette is linked to GFP, expression of IL-17A was monitored by detection of GFP. Actin controlled equal loading. *Right:* Gene expression analysis in mKCs, isolated from the tails of adult mice. *n* = 3–9, ± SEM. Relative mRNA levels were normalized to *Actin*. *P* values were calculated using 2-tailed Student’s *t* test (*P < 0.05, **P < 0.01, and ***P < 0.001).

Although the analysis of IMQ- and IL-36–treated K14-KO mice implies a key role of keratinocyte-derived IκBζ in acute forms of psoriasis, it is unclear whether depletion of IκBζ can also lead to sustained psoriasis protection in models reflecting a more chronic disease course. Moreover, it remains elusive whether keratinocyte-derived IκBζ expression also promotes the development of psoriasis-related comorbidities. To address these questions, we used mice with a keratinocyte-specific overexpression of IL-17A, which triggers not only skin lesions but also systemic inflammation and other psoriasis-associated comorbidities (17, 18). To this end, we crossed our K14-IκBζ–KO mice to conditional IL17A^ind^-knockin mice, which reveal a keratinocyte-restricted IL-17A overexpression upon excision of a loxP-flanked STOP cassette by a K14-controlled Cre recombinase (17, 18, 27). The resulting K14-IL17A^ind^ *Nfkbiz*–KO mice (K14-IL17A^ind^ KO) are hence characterized by keratinocyte-specific transgene expression of IL-17A and a concurrent deficiency of IκBζ, both under the control of K14 promoter–driven Cre expression (Figure 5A). As littermate controls, we used K14-IL17A^ind^-expressing mice harboring a heterozygous loss of *Nfkbiz* in keratinocytes (K14-IL17A^ind^ Ctrl) or mice lacking K14-Cre expression (Ctrl). K14-IL17A^ind^ Ctrl mice developed psoriatic lesions at around week 10, with a progressive phenotype at week 15 (Figure 5B). Of note, presumably because of the deletion of 1 *Nfkbiz* allele, the disease onset of the K14-IL17A^ind^ Ctrl mice occurred later than in K14-IL17A^ind^ mice harboring 2 *Nfkbiz* wild-type alleles (data not shown), which already have full-blown psoriasis at week 6 (18).

Intriguingly, IL-17A–overexpressing mice harboring a keratinocyte-restricted deletion of IκBζ remained completely healthy (Figure 5B), as especially seen by an overall higher weight gain in adulthood (Figure 5C). We detected similar IL-17A levels in the blood of K14-IL17A^ind^ Ctrl and KO mice, suggesting that differences in IL-17A overexpression were not responsible for the observed psoriasis resistance of the IκBζ-KO mice (Figure 5D). Histological analysis of the skin from K14-IL17A^ind^ Ctrl mice revealed hyperproliferative keratinocytes, as well as a massive infiltration of neutrophils and macrophages, whereas these pathological alterations were completely missing in the skin of IκBζ-deficient K14-IL17A^ind^ mice (Figure 5E and Supplemental Figure 3). Furthermore, whereas psoriasis-related genes, such as *Cxcl2*, *Cxcl1*, and *Il1f6*, were significantly induced in the skin of K14-IL17A^ind^ Ctrl mice, the same genes were expressed neither in skin samples (Figure 5F) nor in keratinocytes isolated from mice harboring an additional deletion of IκBζ in keratinocytes (Figure 5G). Therefore, keratinocyte-specific depletion of IκBζ is sufficient to completely suppress IL-17A–mediated skin inflammation in vivo.

### Keratinocyte-specific depletion of IκBζ prevents IL-17A–dependent systemic inflammation.

**Figure 6:**
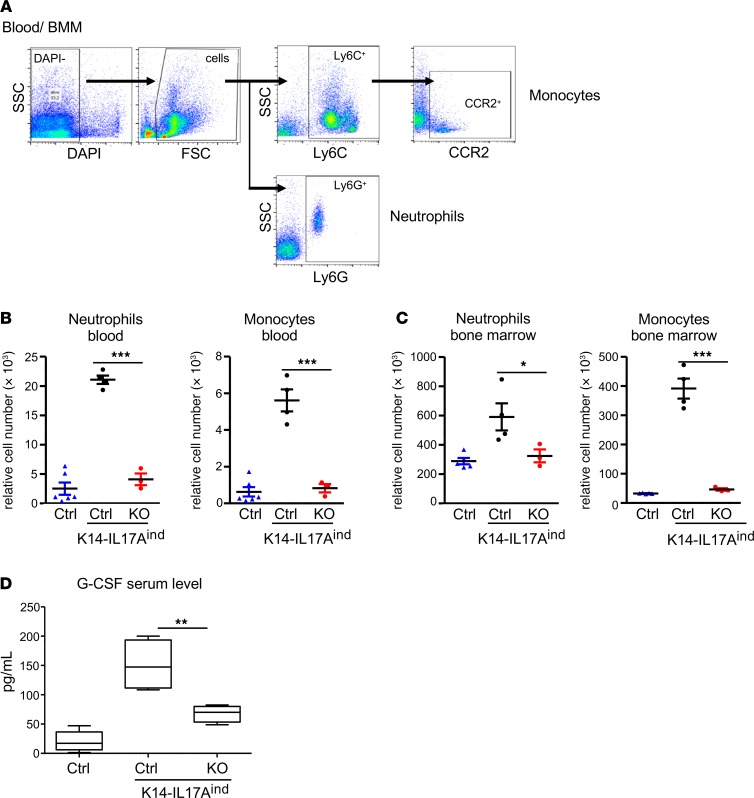
Deficiency of IκBζ in keratinocytes protects against IL-17A–dependent systemic inflammation. (**A**) Gating strategy for the analysis of neutrophils and proinflammatory monocytes from blood and bone marrow. (**B**) Flow cytometry analysis of the numbers of circulating neutrophils (Ly6G^+^) and inflammatory monocytes (Ly6C^+^CCR2^+^) in the blood of IκBζ-proficient and -deficient K14-IL17A^ind^ mice. *n* = 3–6 (mean ± SEM). (**C**) Relative levels of neutrophils (Ly6G^+^) and inflammatory monocytes (Ly6C^+^CCR2^+^) in the bone marrow of K14-IL17A^ind^ mice as detected by flow cytometry. *n* = 3–5 (mean ± SEM). (**D**) Serum levels of G-CSF. The box plots depict the minimum and maximum values (whiskers), the upper and lower quartiles, and the median. The length of the box represents the interquartile range. *n* = 4 (mean ± SEM). *P* values were calculated using 2-tailed Student’s *t* test (*P < 0.05, **P < 0.01, and ***P < 0.001).

Next, we investigated whether the deficiency of IκBζ in keratinocytes also affects systemic inflammation, which is a frequent comorbidity in patients with psoriasis. As reported before (17, 18), mice with a constitutive overexpression of IL-17A in keratinocytes develop a systemic inflammation characterized by increasing numbers of circulating neutrophils and proinflammatory monocytes in the blood, as well as by an upregulated granulo- and monopoiesis in the bone marrow (17, 18). Mechanistically, it is unclear whether this systemic inflammation is a consequence of the psoriasis-like skin inflammation or whether keratinocyte overexpression of IL-17A itself, which was also detectable in the blood, triggers the systemic effects. Interestingly, we found that the deletion of IκBζ in keratinocytes was sufficient to suppress systemic inflammation in mice with a keratinocyte-specific overexpression of IL-17A. Furthermore, we found no increase in the numbers of circulating neutrophils and proinflammatory monocytes in the blood of K14-IL17A^ind^–KO mice, although IL-17A was expressed to the same levels as in IκBζ-proficient littermates (Figure 6, A and B). In line with the normal blood counts of neutrophils and monocytes, K14-IL17A^ind^–KO mice also did not show an upregulated myelopoiesis in their bone marrow (Figure 6C).

The main cytokines driving granulocyte and monocyte differentiation are G-CSF and GM-CSF, which are encoded by *Csf3* and *Csf2*, respectively. Control mice lacking both keratinocyte-specific IL17A overexpression and IκBζ deletion revealed only very low serum levels of GM-CSF, which were, however, significantly increased in K14-IL17A^ind^ Ctrl mice overexpressing IL-17A in the presence of IκBζ (Figure 6D). Interestingly, keratinocyte deletion of IκBζ in the mice with keratinocyte-specific IL-17A transgene expression (K14-IL17A^ind^ mice) resulted in the reduction of GM-CSF to almost normal serum levels. Accordingly, *Csf2* and *Csf3* levels were also increased in the skin and in isolated keratinocytes from K14-IL17A^ind^ Ctrl mice, while expression of both cytokines was suppressed by the lack of IκBζ (Figure 5, F and G). Thus, we propose that IL-17A overexpression in the skin induces — among other proinflammatory genes — IκBζ-mediated expression of *Csf2* and *Csf3*, leading to the secretion of the cytokines into the bloodstream. Subsequently, G-CSF and GM-CSF trigger the generation of neutrophils and monocytes in the bone marrow, leading to their increased mobilization into the bloodstream. Finally, the circulating immune cells infiltrate the skin and other organs, which might be further supported by IκBζ-dependent chemokines (e.g., *Cxcl1*, *Cxcl2*), and result in the establishment of full-blown psoriasis and systemic inflammation.

## Discussion

Recently, *NFKBIZ*, the gene encoding IκBζ, has been identified as a new susceptibility locus in psoriasis (28). In agreement, IκBζ is overexpressed in human psoriatic lesions, whereas global IκBζ-KO mice are completely protected against psoriasis-like skin inflammation in several psoriasis models (7, 8). Although these findings highlight a key role of IκBζ in psoriasis, it remained elusive, so far, how IκBζ expression in the different cell types contributes to psoriasis pathogenesis. Here we describe a key role of keratinocyte-derived IκBζ for the induction of psoriatic skin lesions and, moreover, for systemic inflammation.

Like other atypical IκB proteins, IκBζ can repress but, more importantly, can also induce a specific subset of NF-κB target genes. It is currently thought that IκBζ regulates gene expression mainly at the level of chromatin remodeling. Because IκBζ itself lacks a DNA-binding domain, it requires interaction with the NF-κB subunits p50 or p52 to exert its transcription-enhancing activity. Moreover, IκBζ was found to recruit the epigenetic modifier Tet2 and the SWI/SNF nucleosome remodeling complex to target genes, thereby enhancing promoter accessibility (13, 14). Previously we found that IκBζ expression is strongly induced by IL-17 and IL-36, which both act as critical upstream mediators of psoriasis (7, 8). Interestingly, both cytokines are also downstream targets of IκBζ, underscoring the integral role of IκBζ in the signal transduction of IL-17 and IL-36 and their amplification loops.

Abnormal activation of T_H_17 lymphocytes is considered a major pathogenic driver in psoriasis (3). Moreover, IκBζ was identified as a critical regulator for *Il17a* expression in T_H_17 cells (16), suggesting that IκBζ promotes psoriasis via *Il17a* induction in T cells. Interestingly, we now show that depletion of IκBζ in keratinocytes is sufficient to protect against psoriasis, although these K14-KO mice still display elevated numbers of infiltrating T cells and increased *Il17a* expression in the skin.

Why γδ T cells expand in keratinocyte-specific IκBζ-deficient mice in the absence of IMQ treatment is not entirely clear. An important chemokine for the recruitment of γδ T cells into the skin constitutes *Ccl2* (25, 26). Accordingly, we detected an increased expression of *Ccl2* and its receptor, *Ccr2*, in the skin of K14-KO mice, which might explain the increased presence of γδ T cells in keratinocyte-specific IκBζ-KO mice. Similar to *Ccl2*, expression levels of *Il1b* and *Il23a*, which are required for *Il17a* induction in γδ T cells, were upregulated in untreated K14-KO mice. A previous report detected a similar expansion of T cells in the skin of global IκBζ-KO mice, which was attributed to alterations of the skin microbiome (23). Therefore, keratinocyte-derived IκBζ might not only be important for skin inflammation but also represent a critical regulator of the microbiome, thus explaining why IL-17A–producing γδ T cells expand in the skin of untreated K14-KO animals. Importantly, however, our data clearly show that, despite the increased presence of γδ T cells in K14-KO mice, suppression of IκBζ and IκBζ-dependent target gene expression in keratinocytes is sufficient to fully protect against macrophage and neutrophil infiltration and psoriatic skin inflammation.

Among the many target genes that are induced by IL-17 and IL-36, IκBζ regulates only a small specific subset of the IL-17A– or IL-36 transcriptomes. Overlapping genes of the IL-17 and IL-36 signature seem to be enriched in a group of genes that are not only IκBζ target genes, but also essential pathogenic regulators of psoriasis (8, 29). Among others, important psoriasis-associated target genes of IκBζ include certain chemo- and cytokines (e.g., *Cxcl1*, *Cxcl2*, *Cxcl5*, *Ccl3*, and *Il17c*) as well as antimicrobial proteins, such as S100 calcium-binding proteins (e.g., *S100a9*), β-defensin-2 (*Defb4*), and lipocalin-2 (*Lcn2*). Previous studies revealed an important contribution of keratinocyte-derived CXCL1 and CXCL2 to the development of psoriasis, as these chemokines trigger the infiltration of neutrophils into the skin (30). In agreement, blocking of the CXCR2 receptor using a small-molecule antagonist efficiently blocked IL-36–driven skin inflammation (31). Similarly, we found that neutrophil and macrophage especially infiltration was absent in IMQ- or IL-36–treated K14-Cre *Nfkbiz*-KO mice, along with a lack of expression of *Cxcl1*, *Cxcl2*, or *Cxcl5*. Because we detected direct binding of IκBζ to the promoter regions of the respective genes, we suggest that this specific subset of IκBζ target genes is needed for innate immune cell infiltration and the development of psoriatic plaques.

Our results demonstrate an importance of IκBζ not only in IMQ- and IL-36–triggered psoriasis but also in K14-IL17A^ind^ mice, which overexpress IL-17A specifically in keratinocytes. These mice suffer from a more severe chronic form of psoriasis and, moreover, develop comorbidities and systemic inflammation, which frequently occurs in psoriasis patients. The systemic inflammation of K14-IL17A^ind^ mice was evident by a significantly lower body weight, increased myelopoiesis in the bone marrow, and elevated numbers of circulating neutrophils and monocytes in the blood. On the molecular level, it has been revealed that G-CSF, which is a direct IκBζ target gene (11), contributes to psoriasis by triggering the generation, mobilization, and infiltration of neutrophils (32, 33). Moreover, in cancer patients or heathy volunteers, application of G-CSF was sufficient to trigger psoriasis-like dermatitis (34–37). Similarly, keratinocyte-derived CXCL1 and CXCL2 induce the mobilization of neutrophils from the bone marrow (38, 39), whereas increased generation of proinflammatory monocytes was found to be driven by elevated GM-CSF levels (40). Thus, keratinocyte expression of IκBζ-dependent target genes, including *Cxcl1*, *Cxcl2*, *Csf2*, and *Csf3*, might be responsible not only for psoriatic skin lesions but also for the generation and tissue infiltration of immune cells. These results therefore hint at a potentially critical role of keratinocyte-derived IκBζ in psoriasis-associated comorbidities, which should be further investigated in future studies.

Our data also have therapeutic implications. Whether abnormalities in keratinocytes or immune cells are the primary cause of psoriasis has been debated for a long time. Certainly, an intense crosstalk between different cell types is required for disease development. Although most of the current antipsoriatic therapies target T cell activation, there is increasing evidence for a critical role of keratinocytes. For instance, it was observed that the keratinocyte-specific loss of *Tnfaip3*, encoding the NF-κB inhibitor A20, is sufficient to induce psoriasis-like skin inflammation in mice (41). Likewise, keratinocyte overexpression of constitutively active Stat3, which we have previously identified as a transcriptional regulator of IκBζ (8), triggers spontaneous psoriatic lesions (42). Therefore, topical treatment of keratinocytes might be a rational approach for the therapy of both psoriatic skin lesions and its related comorbidities. Because of the prevalent and conserved function of IκBζ in both IL-17A and IL-36 signaling, targeting of IκBζ expression or function in keratinocytes might become an attractive strategy for an effective long-term psoriasis therapy.

## Methods

### Mice.

Experiments were conducted in accordance with the German law and guidelines of animal care. IMQ-induced psoriasis was carried out with female mice; for IL-36–mediated psoriasis induction, male mice were selected. TAM-inducible IκBζ-KO mice were generated by crossing B6.Cg-Nfkbiz<tm1.1Muta> mice (RIKEN) to B6.129-*Gt(ROSA)26Sor^tm1(cre/ERT2)Tyj^*/J mice (The Jackson Laboratory). For induction of TAM-inducible global IκBζ KO, mice received intraperitoneal injections of 75 mg/kg TAM for 4 days. Control *Nfkbiz^fl/fl^* mice (B6.Cg-Nfkbiz<tm1.1Muta>) were treated in parallel. Keratinocyte-specific IκBζ-KO mice (K14-KO) were generated by crossing B6.Cg.Nfkbiz<tm1.1Muta> mice (RIKEN) to B6N.Cg-Tg(KRT14-cre)1Amc/J mice (stock 018964, The Jackson Laboratory). For the IMQ model, ears of control mice (B6.Cg-Nfkbiz<tm1.1Muta>), global KO, and K14-KO mice were treated with 5 mg Aldara cream (5% imiquimod, 3M Pharmaceuticals) for 7 consecutive days. In the model of inducible IκBζ deletion after IMQ treatment, mice were treated for 2 days with IMQ alone, followed by 4 consecutive days of combined TAM and IMQ treatment. For IL-36–induced dermatitis, mice received intradermal injections into the ears using 1 μg recombinant murine IL-36α (aa 8-160, R&D Systems) or PBS as a control for 5 consecutive days. Ear thickness was measured daily using a precise caliper (IP67/C110T, Kroeplin). Keratinocyte-specific IL-17A–overexpressing mice were generated by crossing B6.Cg-IL17A^ind/ind^ mice (17, 18) to *Nfkbiz^fl/fl^* mice (B6.Cg-Nfkbiz<tm1.1Muta>). The resulting IL17A^ind/+^ *Nfkbiz*^fl/+^ mice were then crossed to K14-Cre *Nfkbiz^fl/fl^* mice, generating Ctrl mice (IL17A^ind/+^ *Nfkbiz^fl/fl^*), keratinocyte-specific IL-17A–overexpressing Ctrl (K14-IL17A^ind^ Ctrl; KRT14 IL17A^ind/+^ *Nfkbiz^fl/+^*), or IκBζ-KO mice (K14-IL17A^ind^ KO; KRT14 IL17A^ind/+^ *Nfkbiz^fl/fl^*). To exclude sex differences, only male mice were used for subsequent analyses. Skin lesions were scored using a cumulative PASI score that describes the degree of scaling, erythema, and percentage of the affected area (0 = no lesions, 1 = very mild, 2 = mild, 3 = intermediate, 4 = severe, and 5 = very severe). IL-17A–overexpressing mice developed skin lesions starting at weeks 8–10 with disease progression up to week 15. At week 15, all mice were sacrificed and analyzed.

### Isolation of murine keratinocytes.

Cells were isolated from 8- to 10-week-old animals by cutting off their tail after cervical dislocation and separation of skin from the muscle. The skin was incubated overnight at 4°C in keratinocyte-SFM medium (Gibco, Thermo Fisher Scientific) supplemented with 50 μg/mL dispase (17105-041, Gibco, Thermo Fisher Scientific). The next day, the epidermis was separated from the dermis and subsequently incubated for 15 minutes in 0.05% trypsin-EDTA (25300-054, Gibco, Thermo Fisher Scientific) solution at room temperature (RT). Trypsin digestion was stopped by adding RPMI medium supplemented with 10% FCS, followed by gentle washout of single-cell keratinocytes. Cells were transferred to a 100-μm cell strainer and collected in a 50-mL Falcon tube. After centrifugation at 180 *g* for 5 minutes, keratinocytes were resuspended in keratinocyte-SFM medium supplemented with 0.05 M CaCl_2_ and seeded on collagen type I–coated plates (A10644-01, Gibco, Thermo Fisher Scientific). After reaching confluence, keratinocytes were stimulated with 100 ng/mL recombinant murine IL-36α for 1.5 hours.

### Histology.

Tissue was fixed overnight with 10% formaldehyde solution (Carl Roth, A146.5). After dehydration and paraffin embedding, 5-μm sections were prepared. Antigen retrieval was performed with 1 mM EDTA, pH 8 (for MPO detection) or 10 mM citrate buffer, pH 6.0 (for F4/80 staining), at 95°C for 20 minutes. Afterward, endogenous peroxidase activity was quenched by incubation of the slides in 5% H_2_O_2_ for 10 minutes. After blocking with 5% normal horse or goat serum (in TBS/0.1% Tween-20 [TBST]), primary antibodies were incubated overnight at 4°C (anti-MPO, AF3667, R&D Systems, 1:200; F4/80, 70076, Cell Signaling Technology, 1:400). The next day, sections were washed with TBST and incubated with secondary HRP-coupled antibody for 30 minutes at RT with SignalStain Boost IHC (8114, Cell Signaling Technology) for F4/80 staining or anti-mouse HRP IgG (A15999, Thermo Fisher Scientific) for MPO detection. Afterward, sections were washed again with TBST and developed using the SignalStain DAB kit (8059, Cell Signaling Technology). DAB substrate turnover was stopped by washing with H_2_O, followed by a short counterstain with hematoxylin and mounting of the slides. For detection of *Nfkbiz* expression by in situ hybridization, RNAScope technology (RNAScope 2.5 HD assay Red, ACDBio) was applied according to the manufacturer’s instructions. Murine *Nfkbiz* was detected with the RNAScope probe Mm-*Nfkbiz* (catalog 806551), which was designed against *Nfkbiz* NM_030612.3, region 742–1642 bp. Human *NFKBIZ* was detected with the RNAScope probe Hs-NFKBIZ (catalog 497851).

### Flow cytometry.

For analysis of the infiltrating immune cell subpopulations, ears of control and IMQ-treated mice were cut into small pieces and incubated with 300 μg/mL liberase (0540102001, Roche) and 50 U/mL DNAse I (EN0523, Thermo Fisher Scientific) in 5% FCS in RPMI medium for 2 hours at 37°C. Afterward cells were passed through a cell strainer (100 μm) to obtain a single-cell suspension. After cell counting, 10^5^ cells were treated with Fc-Block (BUF041, Bio-Rad) for 15 minutes and surface stained with the following mouse-specific antibodies from BioLegend: anti-CD45 APC/Cy7 (catalog 110715), anti-CD45 PerCP (catalog 103129), anti-CD3ε PerCP (catalog 100325), anti-Ly6G PE (catalog 127607), F4/80 APC (catalog 123115), anti-CD45 FITC (catalog 103108), anti-Ly6C APC (catalog 128016), anti-γδTCR FITC (catalog 107503), and anti–IL-22 APC (catalog 516409). Anti-mouse αβTCR Pacific Blue (catalog HM3628) was purchased from Invitrogen and anti–IL-17A PE (catalog eBio17B7) from eBioscience. Intracellular staining for IL-17A and IL-22 in T cell subsets was performed with BD Fixation/Permeabilization Solution Kit (catalog 554714, BD Biosciences), according to the manufacturer’s manual. Prior to analysis, cells were treated with PMA/ionomycin and BD Golgi-Stop (catalog 554724) containing monensin for 4 hours. Acquisition was performed with the LSRII flow cytometer (Becton Dickinson), and live single cells were gated using FlowJo (Tree Star Inc.) software. For neutrophil and monocyte quantification, live cells were gated using DAPI.

### RNA extraction and quantitative PCR.

Total RNA was extracted with QIAzol (79309, QIAGEN) according to the manufacturer’s instructions. Contaminating genomic DNA was removed by treatment with DNAse I (EN0523, Thermo Fisher Scientific) in the presence of Ribonuclease Inhibitor (EO0381, Thermo Fisher Scientific). For cDNA synthesis, 1 μg total RNA from mouse keratinocytes was reverse-transcribed using random hexamer primers (SO142, Thermo Fisher Scientific) and Revert Aid reverse transcriptase (EP0441, Thermo Fisher Scientific). For reverse transcription of RNA from tissue, cDNA was synthesized from 4 μg total RNA using oligo-dT primer (SO132, Thermo Fisher Scientific) and Revert Aid reverse transcriptase. cDNA reaction was performed for 1 hour at 42°C. Relative gene expression was quantified by real-time PCR using the Green master mix from Genaxxon (M3023) and self-designed primers (Supplemental Table 1). Real-time PCR analysis was performed on a Light Cycler 480 II system (Roche) using the following PCR conditions: initial denaturation 15 minutes at 95°C, followed by 40 cycles of 95°C for 15 seconds and 60°C for 45 seconds. Relative mRNA levels were calculated by normalization to the reference gene *Actin* using the 2^-ΔΔCt^ method.

### ChIP.

ChIP was basically done as described (8). In brief, chromatin from mouse keratinocytes was prepared by cross-linking with 1% formaldehyde (4979.1, Carl Roth) for 10 minutes at RT, followed by the addition of 0.25 M glycine to stop the reaction. After washing with PBS, cells were cross-linked for a second time using 2 mM di(*N*-succinimidyl) glutarate (sc285455, Santa Cruz Biotechnology) for 45 minutes at RT. Afterward cells were lysed and sonicated in the presence of 0.3% SDS using a bioruptor (Diagenode). After centrifugation (5 minutes, 18,000 *g*) at 4°C to remove unsheared chromatin and cell debris, chromatin was incubated with G protein–coupled Dynabeads (10004D, Invitrogen) and 5 μL rabbit anti-IκBζ antibody (made in-house) or 2 μg rabbit anti-IgG control antibody (ab46540, Abcam) overnight at 4°C. The promoter region of *Mb* served as an internal negative control (forward: 5′-CTCTGCTCCTTTGCCACAAC-3′, reverse: 5′-GAGTGCTCTTCGGGTTTCAG-3′). ChIP primers for IκBζ target genes were designed for the respective promoter regions corresponding to transcription factor–bound sites (as marked by DNAse I–insensitive regions in the UCSC genome browser). The following primers were used: *Csf3* (forward: 5′-TGGCTGGAAGAGAGGAAGAG-3′, reverse: 5′-TTGTGAAATCGGGGAATCTC-3′), *Cxcl1* (forward: 5′-GTTCCAGCACTCCAGACTCC-3′, reverse: 5′-AGTGGCGAGACCTACCTGTG-3′), *S100a9* (forward: 5′-GCAGGAAATGTTCACACAGC-3′, reverse: 5′-TTGGATGGAAGGGAAGTGAG-3′), *Cxcl2* (forward: 5′-CGCAGACATCACTTCCTTCC-3′, reverse: 5′-AGCTGCCTGCCTCATTCTAC-3′), *Defb4* (forward: 5′-TCCTAAGCCTGTTGCCAGAC-3′, reverse: 5′-GATTTCCTCCTGCACTGCTC-3′), *Il1f6* (forward: 5′-TCTGCTGAAATGTGGACAGG-3′, reverse: 5′-ACCGCAAGTTCTGACCAAAG-3′), and *Il1f9* (forward: 5′-CCTGAACTTCCCAGAAGCAC-3′, reverse: 5′-CTACCAGAGGCACCAGCTTC-3′). Quantitative PCR reaction was performed using the Maxima SYBR Green Master Mix (K0221, Thermo Fisher) under the same conditions as described for the gene expression analysis.

### Western blot analysis.

Cells were washed with PBS and resuspended in lysis buffer containing 20 mM Tris-HCl at pH 7.5, 150 mM NaCl, 1% Triton X-100, 1 mM Na_2_EDTA, 1 mM EGTA, 1 mM β-glycerophosphate, 2 M urea, and 1× protease inhibitor cocktail (Roche). After incubation for 10 minutes on ice, samples were briefly sonicated to disrupt DNA–protein complexes. Afterward, samples were separated by SDS-PAGE and transferred to a nitrocellulose membrane. The following antibodies were used: anti-mouse IκBζ (made in-house, raised against murine peptides CSAPGSPGSDSSDFSS and CLHIRSHKQKASGQ), anti–β-actin (Cell Signaling Technology, catalog 3700), anti-mouse H3 (Cell Signaling Technology, catalog 3638), and anti-GFP (Santa Cruz Biotechnology, catalog sc-9996).

### ELISA.

Quantification of IL-17A and G-CSF levels was performed in serum samples using commercial kits (DuoSet mouse G-CSF ELISA, DY414-05, R&D Systems; mouse IL-17A ELISA, 432504, BioLegend).

### Statistics.

Results are represented as the mean ± SEM. Significance was calculated using a 2-tailed Student’s *t* test. Significance is depicted as asterisks (*P < 0.05, **P < 0.01, and ***P < 0.001).

### Study approval.

All animal experiments were approved by the Regierungspräsidium Tübingen, Germany (IB 3/16, IB 2/17, and IB 1/18). The human psoriasis skin samples were derived from the Department of Dermatology, Heidelberg University Hospital. Experiments were approved by a local ethical committee of the Heidelberg Hospital University in Heidelberg, Germany.

## Author contributions

SL, AM, JL, CR, and PB performed experiments and data analysis. AW and FCK developed the K14-IL-17A^ind^ mice and helped in designing experiments using this mouse model. KS donated human psoriasis skin samples and helped in designing the experiments. DK and SH designed the experiments. SH, KSO, and DK wrote the manuscript.

## Supplementary Material

Supplemental data

## Version 1 10/17/2019

In-Press Preview

## Version 2 11/14/2019

Electronic publication
