# Supplementary material for: Keratinocyte-derived IκBζ drives psoriasis and associated systemic inflammation
Source: JCI Insight. 2019 Nov 14;4(22):e130835. doi: 10.1172/jci.insight.130835 (PMC6948851; doi:10.1172/jci.insight.130835)
Supplement: Supplemental data [file jciinsight-4-130835-s174.pdf]

Supplementary information

Keratinocyte-derived IκBζ drives psoriasis and associated systemic inflammation

Sebastian Lorscheid[1], Anne Müller[1], Jessica Löffler[1], Claudia Resch[1], Philip Bucher[1], Florian C. Kurschus[2,3], Ari Waisman[3], Knut Schäkel[2], Stephan Hailfinger[1,4], Klaus Schulze-Osthoff[1,4,5], Daniela Kramer[1,6]

[1] Interfaculty Institute for Biochemistry, University of Tübingen, Hoppe-Seyler-Strasse 4, 72076 Tübingen, Germany

[2]Department of Dermatology, Heidelberg University Hospital, 69120 Heidelberg, Germany

[3]Institute for Molecular Medicine, University Medical Center of the Johannes Gutenberg-University Mainz, Mainz, Germany

[4] Cluster of Excellence iFIT (EXC 2180) "Image-Guided and Functionally Instructed Tumor Therapies", University of Tübingen, Germany

[5] German Cancer Consortium (DKTK) and German Cancer Research Center (DKFZ), 69120 Heidelberg, Germany

[6] To whom correspondence should be addressed: daniela.kramer@uni-tuebingen.de

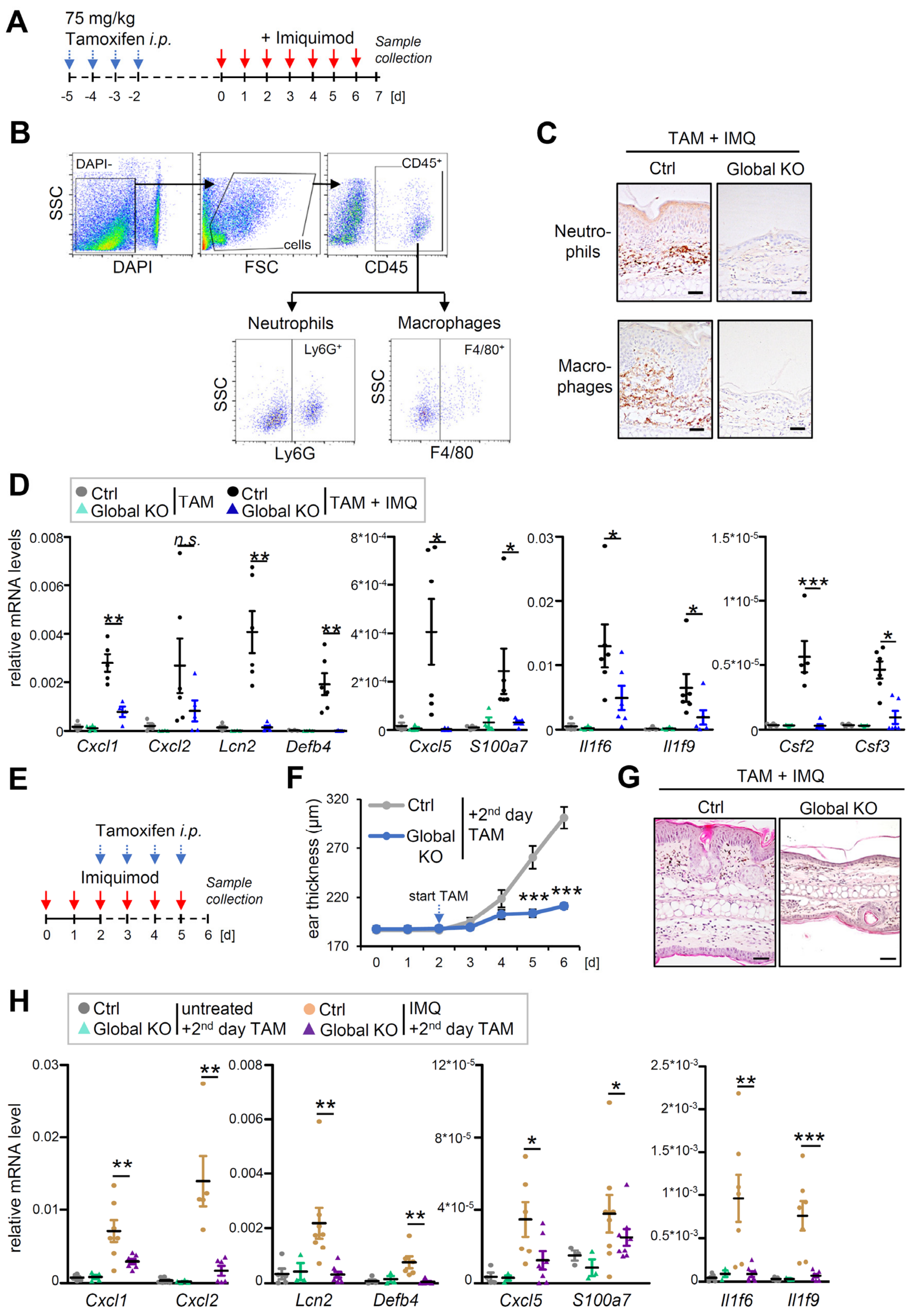

Supplemental Figure 1. Extended phenotype analysis of imiquimod-treated K14-cre *Nfkbiz* KO mice. (A) Treatment scheme for the induction of global *Nfkbiz* deletion using tamoxifen (TAM) injections (*i.p.*) prior to IMQ treatment. **(B)** Gating strategy for the

quantification of neutrophil and macrophage numbers in IMQ-treated ears. **(C)** IHC staining of neutrophils (MPO staining) and macrophages (F4/80 staining) in the ears of IMQ-treated global *Nfkbiz* KO mice. Bars: 40 µm. **(D)** Gene expression analysis of skin samples from 7 d IMQ-treated, tamoxifen (TAM)-inducible global *Nfkbiz* KO mice. Relative mRNA levels were normalized to *Actin*. n = 4 for untreated mice, n = 6 for IMQ-treated mice ± SEM. **(E)** Scheme of IMQ and tamoxifen treatment in order to analyze effects of a delayed global *Nfkbiz* deletion in mice on psoriasis-related gene expression and disease progression. **(F)** Measurement of the ear thickness from mice treated as in (E). **(G)** H&E staining from ears of IMQ-treated control and global (*Nfkbiz*) KO mice at day 7. Mice received tamoxifen treatment 2 days delayed, as depicted in (E). Bars: 40 µm. **(H)** Gene expression analysis from skin samples of mice that were treated as in (E), similar analysis as in (D). n = 3-8 samples per group ± SEM. P-values were calculated using the Student's t-test. (*$p < 0.05$, **$p < 0.01$, ***$p < 0.001$).

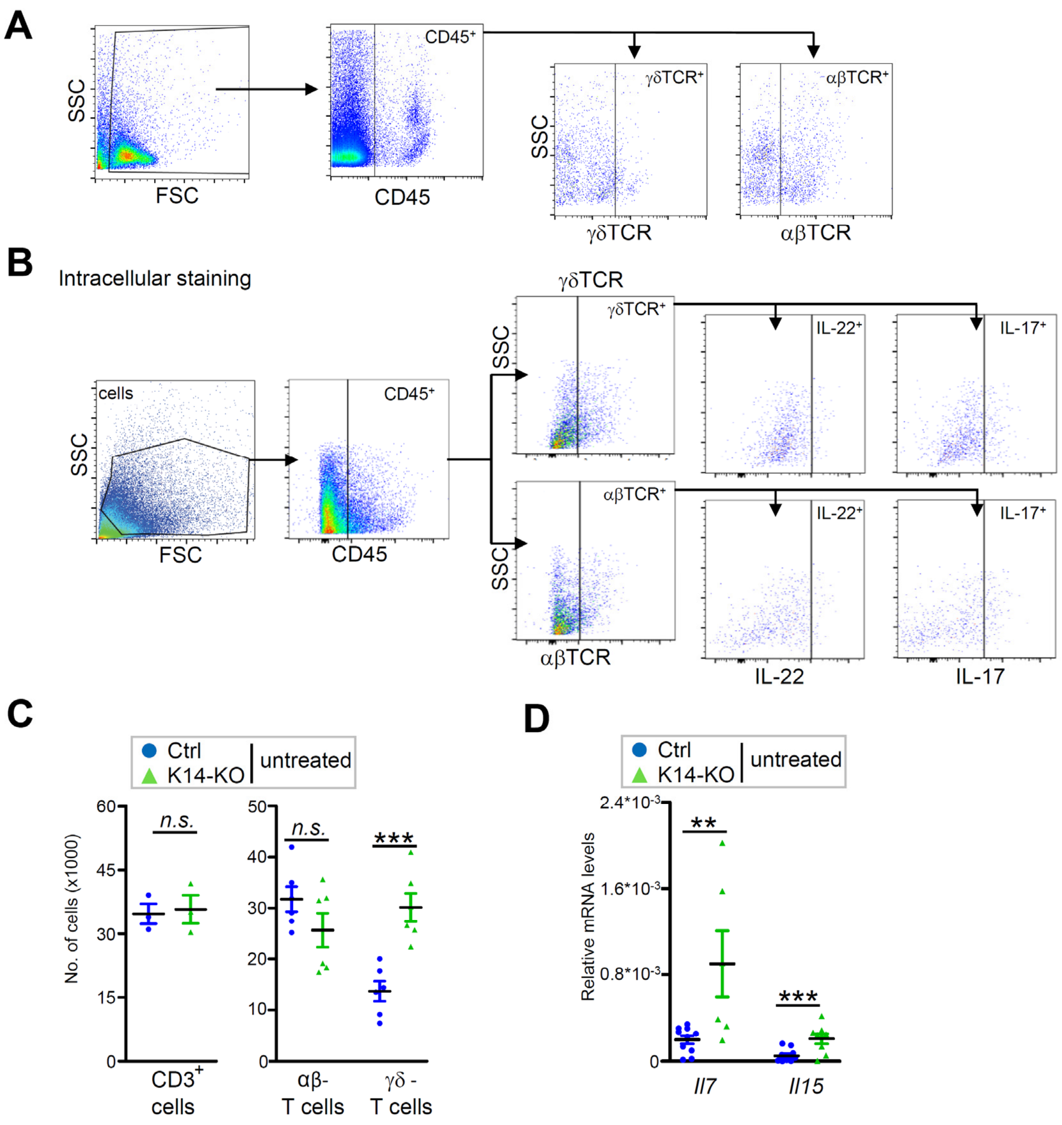

Supplemental Figure 2. Extended analysis of skin-infiltrating T cells in untreated and IMQ-treated K14-cre *Nfkbiz* KO mice. (A) Gating strategy for αβ and γδ T cells from ears of untreated and IMQ-treated mice. **(B)** Gating strategy for intracellular staining of IL-22 and IL-17A in αβ and γδ T cells from IMQ-treated Ctrl and K14-KO mice. Note that gate settings were slightly different as in (A) due to the fixation and permeabilization of the cells. **(C)** Flow cytometry analysis of T-cell subsets in the ears of untreated Ctrl and K14-KO mice. T-cell subsets were detected as $CD45^+$ and either $CD3^+$, $\alpha\beta TCR^+$ or $\gamma\delta TCR^+$ cells. Shown are the mean values of 3-6 ears per group ± SEM. **(D)** Gene expression analysis of *Il7* and *Il15* in untreated Ctrl and K14-KO mice. Relative mRNA levels were normalized to *Actin*. n = 6 ± SEM. P-values were calculated using the Student's t-test. (*p < 0.05, **p < 0.01, ***p < 0.001).

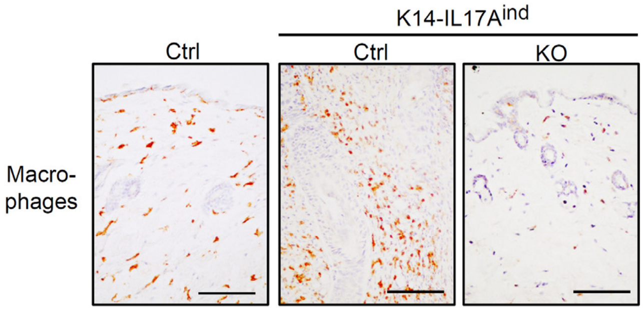

Supplemental Figure 3. Macrophage infiltration into the skin of K14-IL17A^{ind} IκBζ KO mice. Detection of macrophages by F4/80 staining in skin sections of 15-week-old K14-IL17A^{ind} mice with heterozygous or homozygous deletion of IκBζ.

Table S1. List of gene expression primer.

Primer	forward	reverse
Actin	AGGAGTACGATGAGTCCGGC	GGTGTAAAACGCAGCTCAGTA
Ccl17	AATGTAGGCCGAGAGTGCTG	ACAGTCAGAAACACGATGGC
Ccl2	CTGGAGCATCCACGTGTTGG	CCCATTCCTTCTTGGGGTCAG
Ccr4	AGCCTGGTTACAAGCGTAGAG	GAAAGCCAAACTGCACGGAC
Ccr6	CCTCACATTCTTAGGACTGGAGC	GGCAATCAGAGCTCTCGGA
Csf2	TCACGTTGAATGAAGAGGTAGAA G	ACTTGTGTTTCACAGTCCGTTTC
Csf3	ATCCATGGCTCAACTTTCTGC	GCTGCAGGGCCATTAGCTTC
Cxcl1	ACGTGTTGACGCTTCCCTTG	TCCTTTGAACGTCTCTGTCCC
Cxcl2	CGCCCAGACAGAAGTCATAGC	CTTTGGTTCTTCCGTTGAGGG
Cxcl5	CCCTACGGTGGAAGTCATAGC	GAACACTGGCCGTTCTTTCC
Defb4	GGTGCTGCTGTCTCCACTTG	TATTCATCTTGCTGGTTCTTCGTC
Il15	TGCAGTGCATCTCCTTACGC	GTGGATTCTTTCCTGACCTCTCTG
Il17a	GCCCTCAGACTACCTCAACC	TTCCCTCCGCATTGACACAG
Il19	TGTGGACATGCGCCTCATAG	GCAGGTTGTTGGTCATGCAG
Il1b	AGCTGAAAGCTCTCCACCTC	GCTTGGGATCCACACTCTCC
Il1f6	GCCTGTTCTGCACAAAGGATG	ACAGCGATGAACCAACCAGG
Il1f9	GTCAGCGTGACTATCCTCCC	TGGCTTCATTGGCTCAGGG
Il20	TTGGACTGTTCTCCGCTGTG	ATCTTCAGCTTGCACACTATCC
Il22	CCTACATGCAGGAGGTGGTG	CCCAATCGCCTTGATCTCTCC
Il23a	CAGCTCTCTCGGAATCTCTGC	TGTCCTTGAGTCCTTGTGGG
Il7	ATTATGGGTGGTGAGAGCCG	AAAGAAACATGGAACATGGTCTGC
Lcn2	AATGTCACCTCCATCCTGGTC	ACTGGTTGTAGTCCGTGGTG
Nfkbiz	AACTCGCCAAGAGACCAGTG	AGAGCCACTGACTTGGAACG
S100a 7	TCTGCTCTTGGATAGTGTGCC	TGATGTAGTATGGCTGCCTGC
S100a 9	AATGGTGGAAGCACAGTTGG	CTGGTTTGTGTCCAGGTCCTC
